# Divergent Allele Advantage Provides a Quantitative Model for Maintaining Alleles with a Wide Range of Intrinsic Merits

**DOI:** 10.1534/genetics.119.302022

**Published:** 2019-04-08

**Authors:** Thorsten Stefan, Louise Matthews, Joaquin M. Prada, Colette Mair, Richard Reeve, Michael J. Stear

**Affiliations:** * Boyd Orr Centre for Population and Ecosystem Health, Institute of Biodiversity, Animal Health and Comparative Medicine, College of Medical, Veterinary and Life Sciences, University of Glasgow, G12 8QQ, United Kingdom; † Institute of Applied Mathematics and Statistics, University of Hohenheim, 70593 Stuttgart, Germany

**Keywords:** selection, major histocompatibility complex, divergent allele advantage, modeling, evolution

## Abstract

The Major Histocompatibility Complex (MHC) is the most genetically diverse region of the genome in most vertebrates. Some form of balancing selection is necessary to account for the extreme diversity, but the precise mechanism of balancing selection is unknown. Due to the way MHC molecules determine immune recognition, overdominance (also referred to as heterozygote advantage) has been suggested as the main driving force behind this unrivalled diversity. However, both theoretical results and simulation models have shown that overdominance in its classical form cannot maintain large numbers of alleles unless all alleles confer unrealistically similar levels of fitness. There is increasing evidence that heterozygotes containing genetically divergent alleles allow for broader antigen presentation to immune cells, providing a selective mechanism for MHC polymorphism. By framing competing models of overdominance within a general framework, we show that a model based on Divergent Allele Advantage (DAA) provides a superior mechanism for maintaining alleles with a wide range of intrinsic merits, as intrinsically less-fit MHC alleles that are more divergent can survive under DAA. Specifically, our results demonstrate that a quantitative mechanism built from the DAA hypothesis is able to maintain polymorphism in the MHC. Applying such a model to both livestock breeding and conservation could provide a better way of identifying superior heterozygotes, and quantifying the advantages of genetic diversity at the MHC.

## Abstract (precis)

A striking feature of the antigen coding genes of the Major Histocompatibility Complex (MHC) is their genetic diversity. However, the exact mechanisms maintaining this diversity remain elusive. Modelling indicates that Divergent...

A striking feature of the antigen-coding genes of the Major Histocompatibility Complex (MHC) is their extreme genetic diversity (Hedrick 1994). While some form of balancing selection (defined as selection that actively maintains the allelic polymorphism) is necessary to maintain MHC diversity (Hedrick and Thomson 1983), the precise mechanism is unclear. Identifying the underlying mechanisms of MHC polymorphism would answer one of the major questions in immunogenetics and bring substantial benefits to areas as different as precision medicine (where treatment could be tailored by incorporating knowledge about epitopes not recognized by any allele), selective breeding (Stear *et al.* 2005), and conservation genetics (Sommer 2005).

Supported by the important role that the MHC plays in immune recognition (Doherty and Zinkernagel 1975) and the association of MHC genes with many different diseases (Lechler and Warrens 2000), multiple research teams have argued that pathogen-mediated selection influences MHC diversity at MHC loci (Doherty and Zinkernagel 1975; Radwan *et al.* 2010; Spurgin and Richardson 2010). The three main hypotheses for balancing selection on the MHC mediated by pathogens are overdominance (Doherty and Zinkernagel 1975), rare allele advantage (Wright and Dobzhansky 1946; Slade and McCallum 1992), also referred to as (negative) frequency-dependent selection (selection where the fitness of a genotype is negatively correlated with the frequency of the alleles it carries, which can result in a dynamic polymorphism with allele frequencies increasing and decreasing in a cyclical manner), and selection that varies in time and space (Hill *et al.* 1991).

Although considerable amounts of data from nonmodel species in natural populations are available (Schad *et al.* 2005; Piertney and Oliver 2006; Dionne *et al.* 2009; Wayne *et al.* 2013; Grossen *et al.* 2014), the empirical evidence is inconclusive (Bernatchez and Landry 2003; Sommer 2005). This may be attributed to the fact that the extreme genetic diversity at the MHC reduces the statistical power of experimental comparisons (Stear *et al.* 2007): as the number of possible genotypes (comparison groups) becomes large and the frequencies of individual genotypes decrease, statistical power drops. Furthermore, observed allele frequencies, frequency changes, and heterozygosity are potentially compatible with more than one mechanism of pathogen-mediated selection (Spurgin and Richardson 2010). In addition, neutrality tests based on the departures of allele frequency distributions from neutrality are problematic when trying to infer balancing selection on MHC genes, as negative frequency-dependent selection in many cases does not give significantly different results to neutral expectations (Ejsmond *et al.* 2010).

A number of simulation studies (see below) have addressed the question of whether overdominance can maintain allele diversity, but differences in model assumptions and sophistication have resulted in authors arriving at differing conclusions. Maruyama and Nei (1981) found that overdominant selection could substantially increase mean heterozygosity compared with a neutral model, which led them to conclude that overdominance has the potential to explain MHC diversity. They make a distinction between *symmetric* overdominance, in which case all alleles are assumed to confer identical fitness, and *asymmetric* overdominance (AO), which allows alleles to differ in the fitness they confer. Others—such as Gillespie (1977), Lewontin *et al.* (1978), and De Boer *et al.* (2004)—rejected AO because large numbers of alleles could only coexist when alleles showed unrealistically small variation in the level of fitness they conferred. When Spencer and Marks (1988, 1992) extended these models to incorporate mutation, the predicted MHC diversity increased, but only to ∼30–40 alleles, remaining well below observed values for some MHC loci, where the numbers of alleles can exceed 100 for the most polymorphic locus in a number of mammalian species, including cattle (BoLA-DRB3) and sheep (Ovar-DRB1) species, and a large number of nonhuman primate species (EMBL-EBI 2018).

Divergent allele advantage (DAA) (Wakeland *et al.* 1990) is a variant of overdominance that postulates that large numbers of alleles can be maintained as a result of divergent alleles recognizing different peptides. The term DAA was first coined by Wakeland *et al.* (1990), when they examined diversity of MHC alleles in the genus *Mus*. They concluded that MHC class II alleles found in natural mouse populations can be grouped into ancient allelic lineages with substantial divergence between them. This provides individuals with two alleles from different (and thus strongly divergent) lineages with better coverage of the “immune response void,” *i.e.*, better protection against pathogens. A number of studies have made empirical observations consistent with DAA (She *et al.* 1990, 1991; Dorak *et al.* 2002; Radwan *et al.* 2007; Mona *et al.* 2008), with further evidence supporting this hypothesis continuing to emerge (Lenz 2011; Eizaguirre *et al.* 2012; Froeschke and Sommer 2012; Bitarello *et al.* 2016; Seifertová *et al.* 2016; Marmesat *et al.* 2017), most recently showing that heterozygotes with divergent alleles are maintained in the human population and recognize the signatures of greater numbers of peptides than genetically closer alleles (Pierini and Lenz 2018). These results support the hypothesis that divergent alleles should be preferentially maintained in the population, but what is still lacking is a quantitative model demonstrating that this mechanism will result in higher allelic diversity and a wide range of allelic fitnesses when compared to traditional AO models.

Until now, few attempts have been made to build a model based on the DAA hypothesis. Satta (1997) compared a model that counted the differences between codons in the peptide-binding region (without referring directly to DAA) to a symmetric overdominance model, but found the latter to be closer to observed patterns. However, this appears to be driven by the larger number of alleles maintained by a symmetric model. Lau *et al.* (2015) found that a series of models based on DAA, while assuming identical allelic fitness, could only maintain levels of genetic diversity in human leukocyte antigen alleles similar to those in Spencer and Marks (1992), described above, and further that this was only achieved by adding a symmetric overdominance component.

Here, we address these concerns by proposing a novel AO model that captures the notion of enhanced fitness for genotypes with a large number of differences between alleles in the antigen-binding site. Greater differences are assumed to result in better protection against pathogens, since dissimilar alleles have less “overlap” in peptide recognition and therefore recognize a greater variety of pathogen epitopes (Wakeland *et al.* 1990), an idea that forms the basis of the DAA hypothesis and underlies our simple model.

We address discrepancies in approach and findings by classifying existing overdominance models within a common mathematical and computational framework, and systematically examine their weaknesses in maintaining MHC diversity. We then present this novel DAA-based model and demonstrate that it has the potential to maintain a greater number of alleles with a wider variation in allelic fitness, providing quantitative support for this variant of overdominance potentially being a key mechanism for the maintenance of MHC diversity.

## Materials and Methods

### The single-locus model

We first describe the classic single-locus model that provides the framework for comparison of alternative overdominance models.

We considered an effectively infinite vertebrate population with discrete, nonoverlapping generations and random mating, and examined a single autosomal locus with alleles $A_{i}\left(i=1,2,\cdots ,k\right)$, at frequencies $p_{i}\;\left(where\sum_{i=1}^{k}p_{i}=1\right)$. Assuming Hardy–Weinberg equilibrium, an individual with alleles $A_{i}$ and $A_{j}$ occurs at frequency $2p_{i}p_{j}$ for i<j and $p_{i}{}^{2}$ for i=j. Since our focus is on the pathogen-mediated processes in a host–pathogen system, we considered the fitness $f_{ij}$ of a genotype $A_{i}A_{j}$ to be the effectiveness with which the host immune system recognizes different pathogens, which consequently determines the relative frequency of that genotype in the next generation. A fitness of 0 corresponded to genotypes that are not viable, whereas a fitness of 1 corresponded to genotypes that are fully protected against all pathogens.

The classical single-locus multi-allele viability model (Crow and Kimura 1970) specifies the allele proportions in the next generation given the current generation. This can be written, with vectors and matrices in bold text throughout, as (Karlin and Lessard 1984; Nagylaki 1992):

$$p_{i}{}^{\left(t+1\right)}=p_{i}{}^{\left(t\right)}\cdot \frac{w_{i}{}^{\left(m\right)}\left(p^{\left(t\right)}\right)}{\bar{w}\left(p^{\left(t\right)}\right)}\left(i=1,2,\cdots ,k\right)$$ (1)

Here, $p^{\left(t\right)}$ and $p^{\left(t+1\right)}$ are the proportions of all alleles in the system at times t and t+1, $\bar{w}\left(p^{\left(t\right)}\right)$ is the population fitness at time t, and $w^{\left(m\right)}\left(p^{\left(t\right)}\right)$ is the marginal fitness of an allele, which is defined as the average fitness of the genotypes in which it is present, weighted by the proportion of each genotype in the population:

$$w_{i}{}^{\left(m\right)}\left(p^{\left(t\right)}\right)=\sum_{j=1}^{k}f_{ij}p_{j}{}^{\left(t\right)}={\left(Fp_{t}\right)}_{i}\left(i=1,2,\cdots ,k\right)$$ (2)

where $F={\left(f_{ij}\right)}_{1\leq i,j\leq k}$ is the genotype fitness matrix of all genotypes made up of alleles from the set $A_{1},\cdots ,A_{k}$.

The population fitness $\bar{w}\left(p^{\left(t\right)}\right)$ can also be expressed as the weighted mean of the marginal fitness values of all alleles present in the gene pool:

$$\bar{w}\left(p^{\left(t\right)}\right)=\sum_{j=1}^{k}p_{j}{}^{\left(t\right)}\cdot w_{j}{}^{\left(m\right)}\left(p^{\left(t\right)}\right)=\sum_{i=1}^{k}\sum_{j=1}^{k}f_{ij}p_{i}{}^{\left(t\right)}p_{j}{}^{\left(t\right)}$$ (3)

Equation 1 describes a discrete time dynamical system. The equilibrium proportions, $p^{*}$, satisfy

$$w_{i}{}^{\left(m\right)}\left(p^{*}\right)={\left(Fp^{*}\right)}_{i}=\bar{w}\left(p^{*}\right)\left(i=1,2,\cdots ,k\right)$$ (4)

*i.e.*, the marginal fitness of each allele is equal at equilibrium (Lewontin *et al.* 1978; Nagylaki 1992), which corresponds to a stable k-allele system with ${\left(p^{*}\right)}_{i}>0$ for all i.

The equilibrium proportions can therefore be obtained by solving the system of linear equations (Karlin and Lessard 1984; Nagylaki 1992)

Fx=u (5)

where x is the solution vector and u is a vector of ones of length k, eliminating any alleles with nonpositive frequencies (where $x_{i}\leq 0$), and repeating until all remaining alleles had strictly positive values of $x_{i}$. If the resulting principal submatrix $\hat{F}$ of the genotype fitness matrix F (that is restricted to the indices i where $p_{i}>0$) is nonsingular, which ensures global stability of this equilibrium relative to all initial polymorphic states (Karlin and Lessard 1984), then the solution x is normalized, yielding the equilibrium proportions $p^{*}$ where the population fitness $\bar{w}\left(p^{\left(t\right)}\right)$ achieves a strict maximum:

$$p^{*}{}_{i}=\frac{x_{i}}{\sum_{j=1}^{k}x_{j}}\left(i=1,2,\cdots ,k\right)$$ (6)

Otherwise, in the case that $\hat{F}$ is singular, the solution is discarded and the process repeated with a new set of alleles (and therefore, in general, a new F) until this is no longer true, thereby ensuring that all identified equilibria are stable and unique, with the population fitness monotonically increasing until an equilibrium is reached (Karlin and Lessard 1984). This behavior was additionally tested during code validation by running simulations on a time-step basis using Equation 1, starting from an initial state where all initial alleles had equal proportions. These simulations resulted in the same equilibrium as the process using Equation 5 and Equation 6, with the population fitness monotonically increasing until it reached a quasi-equilibrium state where the marginal fitnesses of all persisting alleles took the value of $\bar{w}\left(p^{\left(t=\infty \right)}\right)$ up to 11 decimal places.

### Application to selected overdominance models

We characterized the competing overdominance models in terms of the genotype fitness matrix F, as this matrix fully determines allele equilibrium frequencies. For this, we defined the intrinsic merit $w_{i}$ of an allele $A_{i}$ as the fitness of a homozygote that contains two copies of this allele, *i.e.*, $w_{i}=f_{\mathrm{ii}}$. We further ordered the alleles so that the intrinsic merits of the alleles were nonincreasing $\left(w_{1}\geq w_{2}\geq \cdots \geq w_{k}\right)$.

The genotype fitness matrix F was always assumed to be symmetric, *i.e.*,

$$f_{ji}=f_{ij}\left(\forall i,j\right)$$ (7)

Thus, in its most general form, the genotype fitness matrix F can be written as

$$F=\left(\begin{array}{cc} \begin{array}{cc} w_{1} & f_{12}\\ f_{12} & w_{2} \end{array} & \begin{array}{cc} \cdots & f_{1k}\\ \cdots & f_{2k} \end{array}\\ \begin{array}{cc} \vdots & \vdots \\ f_{1k} & f_{2k} \end{array} & \begin{array}{cc} \ddots & \vdots \\ \cdots & w_{k} \end{array} \end{array}\right)$$ (8)

Although this is a single-locus model, it can be applied to genetic variants ranging from SNP through protein-coding alleles to haplotypes containing multiple protein-coding alleles, so long as recombination is negligible.

### Symmetric overdominance

Under the symmetric overdominance model, all heterozygotes are assumed to have the same fitness, which can be normalized to 1, *i.e.*, $f_{ij}=1$ for i≠j (Robertson 1962). Such a model therefore assumes that heterozygotes are fully protected against every pathogen. If $w_{i}<1$, the model represents overdominance: the heterozygote is always fitter than the corresponding homozygote.

In this model, the k-allele polymorphism $\left\{A_{1},A_{2},\cdots A_{k}\right\}$ is always maintained, irrespective of the intrinsic merits of the alleles $w_{i}$ (Marks and Spencer 1991). Moreover, new alleles are able to invade without displacing any of the k original alleles (De Boer *et al.* 2004); thus, the model accumulates alleles unless stochastic extinction (the loss of an allele from a finite population via random events, especially the death of the last individual carrying this allele) is allowed.

Most commonly, a *fully* symmetric overdominance model (Kimura and Crow 1964; Takahata and Nei 1990; Marks and Spencer 1991; Satta *et al.* 1994; Lau *et al.* 2015) is considered. This is the special case when all alleles have the same intrinsic merit $\left(w_{i}=w_{j}<1\forall i,j\right)$, *i.e.*, all heterozygotes and all homozygotes have equal fitness, with the fitness of the homozygotes lower than that of the heterozygotes (Meyer and Thomson 2001).

As both the general and fully symmetric overdominance model do not allow for allelic loss (*i.e.*, even alleles with very low intrinsic merits persist at equilibrium, contrary to the predictions of the other models discussed), and experimental evidence does not support it (Bronson *et al.* 2013), we disregard it as a plausible mechanism for maintaining divergent alleles.

### AO

Here, we use the term AO to describe a model in which the fitness advantage gained by heterozygotes depends on the fitness of each allele (De Boer *et al.* 2004):

$$f_{ij}=w_{i}+w_{j}-w_{i}\cdot w_{j}\left(i\neq j\right)$$ (9)

This expression captures the combined protective effect of each allele by adding up the intrinsic merits of the alleles, which are derived from the pathogens recognized, and discounting an (average) overlap $w_{i}\cdot w_{j}$ of their contributions, derived from the pathogens that both alleles recognize. Therefore, the advantages or disadvantages of each allele are reinforced in the heterozygote, in a way that only two alleles that both have high intrinsic merits can combine to heterozygotes with a high fitness. In fact, the heterozygote fitnesses are strictly ordered according to the underlying allele-intrinsic merits. Given that $0<w_{k}\leq \cdots \leq w_{1}<1$, then for each off-diagonal element $f_{ij}$ of F

$$\text{max}\left(f_{ii},f_{jj}\right)=\text{max}\left(w_{i},w_{j}\right)<f_{ij}=w_{i}+\left(1-w_{i}\right)\cdot w_{j}<1\left(i\neq j\right)$$ (10)

*i.e.*, heterozygotes are fitter than the corresponding homozygotes and therefore Equation 9 specifies an overdominance model.

In this model, the stability of the k-allele system, *i.e.*, the persistence of all k-alleles at equilibrium, only depends on the intrinsic merits of the alleles (see Equation 9). Here, a threshold value t can be calculated,

$$t=\frac{k-1}{k}\cdot \hat{w}$$ (11)

where $\hat{w}$ is the harmonic mean of $w_{1},\cdots ,w_{k}$. De Boer *et al.* (2004) demonstrated that all k alleles can persist if and only if the intrinsic merits, $w_{i}$, of all alleles are above the threshold value, t. Critically, this implies that, in this model, the intrinsic merits of the alleles have to become more similar the larger k becomes if all k alleles are to persist (De Boer *et al.* 2004).

Alternative formulations for AO behave similarly to the AO model discussed above, as all share a key feature, namely the reinforcement of the intrinsic merits of the two alleles in the heterozygote, such that weaknesses of one allele cannot be compensated by the other allele in the heterozygote.

### DAA

**Figure 1:**
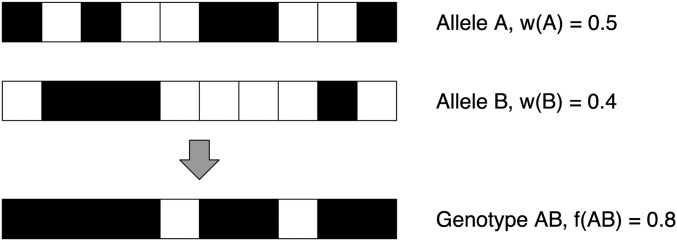
Example calculation of genotype fitness from allele intrinsic merits for the Divergent Allele Advantage model. Black squares represent recognized epitope sets, whereas white squares represented unrecognized epitope sets. In this example, allele A recognizes five epitope sets (out of 10) and therefore has an intrinsic merit of $w_{A}=0.5$. Allele B recognizes four epitope sets and therefore has an intrinsic merit of $w_{B}=0.4$. All epitopes, other than those in positions 5 and 8 (from left), are recognized by one or both alleles. As Major Histocompatibility Complex genes are codominantly expressed (Potts and Slev 1995), *i.e.*, both alleles of a heterozygote are expressed and can therefore present distinctive sets of epitopes, it is sufficient for one of the alleles to recognize an epitope. This results in a genotype fitness $f_{AB}=0.8$ for the heterozygote.

The overdominance models presented above are mathematically tractable but lack an explicit mechanistic basis for the relationship between allele-intrinsic merits and genotype fitnesses. Therefore, we developed a novel model based on the idea of DAA (Wakeland *et al.* 1990). Mathematically, this model can be captured within the same general framework used to describe the traditional overdominance models. The model determines the fitness of a heterozygous genotype from the number of epitopes (antigen parts) recognized by the immune system of an individual of this genotype, which is the union of those recognized by allele A and those recognized by allele B (illustrated in Figure 1).

The key difference between the AO and this DAA model is that overlap is not calculated as an average property of the alleles, but depends on the specific alleles involved. Unlike the AO model, alleles of lower intrinsic merit may combine in a complementary way (with little or no overlap in recognition sites) to form heterozygotes with high fitness in the DAA model, as illustrated by the example below.

### Simple comparison of stability in AO and DAA models

Consider a system of three alleles, with intrinsic merits of $w_{1}=0.8$, $w_{2}=0.7$, and $w_{3}=0.1$. The AO model predicts that such a system is unstable, since the intrinsic merit of allele $A_{3}$, $w_{3}$, lies below the stability threshold value of $\frac{k-1}{k}\cdot \hat{w}$, which in this case is ∼0.158 (see Equation 11). In the DAA model, the outcome depends on the positions of the pathogen-recognition sites.

Assuming the following layout for the pathogen-recognition sites (1s) in the three-allele system $A_{1}$, $A_{2}$, and $A_{3}$,

$$A_{1}0111111110w_{1}=0.8$$

$$A_{2}1111111000w_{2}=0.7$$

$$A_{3}0000000001w_{3}=0.1$$

yields the following genotype fitness matrix F for the DAA model:

$$F=\left(\begin{array}{ccc} 0.8 & 0.9 & 0.9\\ 0.9 & 0.7 & 0.8\\ 0.9 & 0.8 & 0.1 \end{array}\right)$$ (12)

The three alleles have the same intrinsic merits as in the AO formulation, yet the system is stable, giving equilibrium proportions of the alleles $A_{1}$, $A_{2}$, and $A_{3}$ of 65.2, 30.4, and 4.3%, respectively. The behavior of the models differs because the fitness values of the heterozygotes are closer together in the DAA model $\left(f_{12}=0.9,f_{13}=0.9,\text{and}f_{23}=0.8\right)$ than in the AO model $\left(f_{12}=0.94,f_{13}=0.82,\text{and}f_{23}=0.73\right)$, acting to stabilise the system (Lewontin *et al*. 1978). There exist other recognition-site layouts with three alleles of the same intrinsic merits that do not lead to a stable polymorphism in the DAA model. For example, the system:

$$A_{1}0111111110w_{1}=0.8$$

$$A_{2}0111111010w_{2}=0.7$$

$$A_{3}0001000000w_{3}=0.1$$

results in the genotype fitness matrix F:

$$F=\left(\begin{array}{ccc} 0.8 & 0.8 & 0.8\\ 0.8 & 0.7 & 0.7\\ 0.8 & 0.7 & 0.1 \end{array}\right)$$ (13)

In this system, alleles $A_{2}$ and $A_{3}$ will eventually vanish from the population over time, their proportions approaching zero at equilibrium (if the population size is effectively infinite, the proportions of these alleles will still be positive in finite time, whereas in populations of finite size, alleles $A_{2}$ and $A_{3}$ will vanish from the population in finite time due to stochastic extinction), leaving only allele $A_{1}$ at an equilibrium proportion of 100%. This system differs from the previous one in that no heterozygous genotypes exist that are fitter than the homozygous $A_{1}A_{1}$ genotype (the heterozygous genotypes do not recognize more epitopes than the homozygous $A_{1}A_{1}$ genotype), and therefore the highest achievable population fitness is the fitness of the heterozygous $A_{1}A_{1}$ genotype (0.8), which can only be achieved if the proportion of $A_{1}$ is 100%.

These simple examples show clearly that the number of alleles maintained at equilibrium strongly depends on the degree of complementarity of the alleles present in the DAA model.

### Simulation scenarios explored

The AO and DAA models were compared across 80 scenarios (Table 1) that varied in their number of initial alleles $n_{\mathrm{ini}}$, and the minimum and maximum intrinsic merit of any allele $\left(w_{\min},w_{\max}\right)$. The length of the epitope-recognition site sequence $l_{S}$ (*i.e.*, the total number of black and white squares, Figure 1) was then chosen to be sufficiently large to reduce the possibility of multiple alleles having identical intrinsic merits, by ensuring that there were either 2 or 10 (*f*) times as many possible intrinsic merits in the range selected for the scenario as the number of initial alleles.

**Table 1:** Overview of the scenarios explored

Scenarios	$w_{min}$	$w_{max}$	*n_ini_*	*f*
1–5	0	0.1	50, 100, 250, 500, 1000	2
6–10	0	0.1	50, 100, 250, 500, 1000	10
11–15	0.45	0.55	50, 100, 250, 500, 1000	2
16–20	0.45	0.55	50, 100, 250, 500, 1000	10
21–25	0.2	0.6	50, 100, 250, 500, 1000	2
26–30	0.2	0.6	50, 100, 250, 500, 1000	10
31–35	0.3	0.7	50, 100, 250, 500, 1000	2
36–40	0.3	0.7	50, 100, 250, 500, 1000	10
41–45	0.4	0.8	50, 100, 250, 500, 1000	2
46–50	0.4	0.8	50, 100, 250, 500, 1000	10
51–55	0.1	0.9	50, 100, 250, 500, 1000	2
56–60	0.1	0.9	50, 100, 250, 500, 1000	10
61–65	0.0	1.0	50, 100, 250, 500, 1000	2
66–70	0.0	1.0	50, 100, 250, 500, 1000	10
71–75	0.9	1.0	50, 100, 250, 500, 1000	2
76–80	0.9	1.0	50, 100, 250, 500, 1000	10

We chose the scenarios to reflect a wide range of possible biological systems and provide results of general applicability. The minimum and maximum intrinsic merits $w_{min}$ and $w_{max}$ were chosen to capture situations where both are low (scenarios 1–10), high (scenarios 71–80), where $w_{min}$ is low and $w_{max}$ high (scenarios 51–70), or both are intermediate. The initial number of alleles is generally not known, but as > 100 alleles have been found for a number of mammalian species (see above), we also explored larger values for $n_{ini}$, as well as different epitope-recognition site sequence lengths, varying f to cover situations where the individuals are exposed to fewer or more pathogens.

We ran multiple repeats for each scenario (10,000 repeats for 50 and 100 initial alleles, 2000 for 250 initial alleles, 500 for 500 initial alleles, and 100 for 1000 initial alleles, with the numbers reducing for computational reasons). In each repeat, we first drew intrinsic merits for the initial alleles from a uniform distribution between $w_{min}+\varepsilon$ and $w_{max}-\varepsilon$, where $\varepsilon =\frac{w_{max}-w_{min}}{2\cdot n_{ini}}$ (and $\;n_{ini}$ the initial number of alleles), ensuring that alleles with an intrinsic merit of 1 (a “perfect” allele) and 0 (a “useless” allele) could not be selected. For an allele $A_{i}$, we obtained the number of recognition sites $m\left(A_{i}\right)$ by multiplying each intrinsic merit by $l_{S}$ (epitope-recognition site sequence length), so $m\left(A_{i}\right)=l_{s}\cdot w\left(A_{i}\right)=l_{s}\cdot w_{i}$. We then created the recognition-site pattern for that allele by randomly choosing $m\left(A_{i}\right)$ locations among $l_{S}$ sites, assigning these to be black squares and the remainder white. Finally, for each scenario, we calculated the sets of persisting alleles using Equation 5 and Equation 6. We used the same allele-intrinsic merits and epitope-recognition pattern (*i.e.*, the positions of the epitope-recognition sites for each allele), for both the AO and DAA model, to allow paired comparisons.

After running these ∼720,000 distinct simulations varying both the intrinsic merits and the epitope-recognition patterns (the “Random” experiment), we then repeated the 80 scenarios focusing only on variability due to change in the epitope-recognition pattern. In this case (the “Fixed” experiment), we held the intrinsic merits at fixed, evenly spaced values across the range $w_{min}+\varepsilon$ to $w_{max}-\varepsilon$ for each scenario, and only the positions of the epitope-recognition sites were redrawn for each iteration. For example, for scenario 62, the 100 initial alleles had intrinsic merits of 0.005, 0.015 … 0.995. This provided us with multiple (100–10,000 as above) repeats of the same set of initial allele-intrinsic merits, thereby allowing us to highlight a key feature of the DAA concept by exploring the variation of extant allele numbers and intrinsic merit ranges that stems from variability in epitope-recognition patterns alone. The two experiments are referred to below as the Random and Fixed experiments, respectively.

We compared the AO and DAA models in every iteration, scenario, and experiment using three metrics applied to the alleles persisting at equilibrium: the number of alleles maintained $n_{equil}$, the range of intrinsic merits of these alleles $r_{equil}$, and the average overdominance (or heterozygote advantage) $\bar{h}$ (and $h_{i}$ for allele), given by the increase in the average fitness of heterozygotes compared to the average fitness of homozygotes, both at equilibrium, *i.e.*, $\bar{h}=\frac{\bar{w_{het}}-\bar{w_{hom}}}{\bar{w_{hom}}}$ (and $h_{i}=\frac{w_{het,i}-w_{hom,i}}{w_{hom,i}}$). Furthermore, we calculated the average overlap of epitope recognition between a particular allele and all other alleles of the gene pool (Equation 14), for all alleles of the final gene pool for the same experiments and scenarios as above, but with a reduced number of repetitions (500 repeats for 50 initial alleles, 200 for 100 initial alleles, 50 for 250 initial alleles, 20 for 500 initial alleles, and 5 for 1000 initial alleles). This calculation was done in the same way for the AO and DAA model. We defined the average overlap $g_{i}$ for allele $A_{i}$ as a weighted mean of the proportion of epitopes recognized by both alleles relative to the length of the epitope-recognition site sequence $l_{S}$, with the allele proportions $p_{j}$ as weights ($r_{ij}$ is the number of epitopes recognized by alleles i and j):

$$g_{i}=\frac{\sum_{j=1}^{k}p_{j}\cdot g_{ij}}{1-p_{i}},g_{ij}=\frac{r_{ij}}{l_{s}}$$ (14)

### Data availability

The Python code that was used to generate the results can be found at https://zenodo.org/badge/latestdoi/168343566. Supplemental files, including full simulation results for both the effectively infinite population and, in addition, for a population of 1000 individuals are available at Figshare. Supplemental Material, Files S1 and S3 contain mean and maximum number of alleles, and intrinsic merit ranges for both the AO model and the DAA model, and each scenario of the Random experiment for the effectively infinite population and the population of 1000 individuals, respectively. Files S2 and S4 contain mean and maximum number of alleles, and intrinsic merit ranges for both the AO model and the DAA model, and each scenario of the Fixed experiment, again for the effectively infinite population and the population of 1000 individuals, respectively. Files S5 and S6 contain statistics related to the average overlap between alleles, and compare the AO model to the DAA model for the Random experiment and the Fixed experiment, respectively. File S7 lists the approximate frequency of the genotype fitness matrix F being singular for most scenarios, and includes some explanation and interpretation.

Files 01__Random_Experiment__PopSize_Inf.zip and 02__Fixed_Experiment__PopSize_Inf.zip contain the simulation results of the Random and Fixed experiments for the effectively infinite population, while files 03__Random_Experiment__PopSize_1000.zip and 04__Fixed_Experiment__PopSize_1000.zip contain the simulation results of the Random and Fixed experiments for the population of 1000 individuals. Supplemental material available at https://doi.org/10.25386/genetics.7901519.

## Results

**Figure 2:**
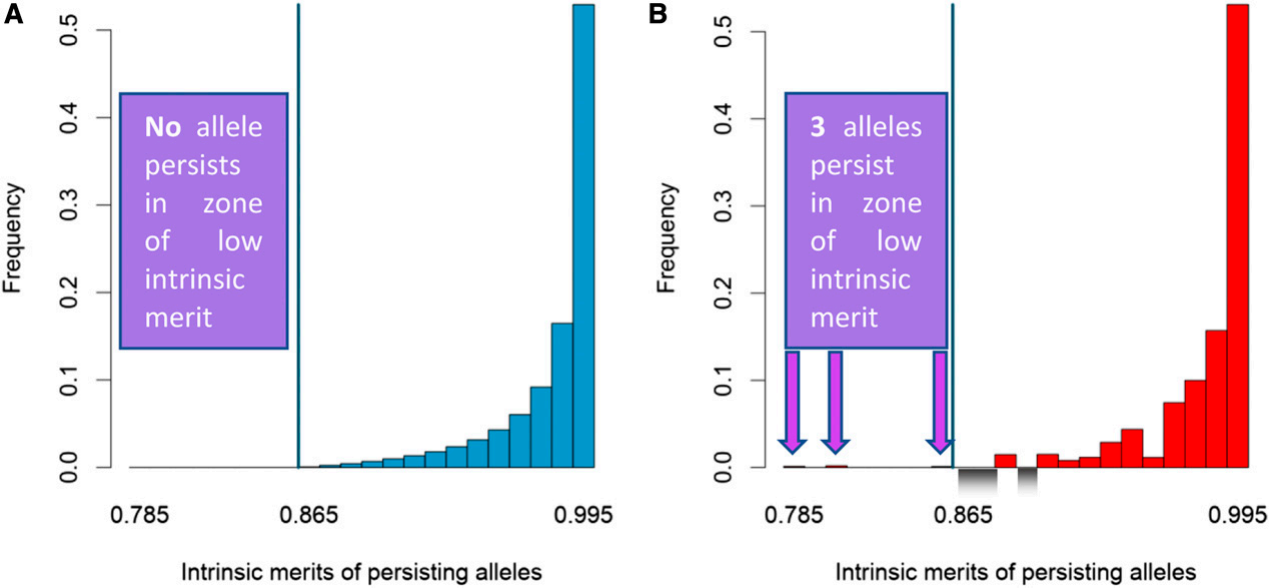
Frequencies and range of persisting alleles at equilibrium. Shown for scenario 62 of the Fixed experiment, defined by the quadruple $\left(w_{min},w_{max},f,n_{ini}\right)=\left(0.0,1.0,2,100\right)$ (see Table 1 and Table S2) for (A) the asymmetric overdominance (AO) and (B) the Divergent Allele Advantage (DAA) model. The distribution in (B) is illustrative as it corresponds to a single, random set of starting alleles. The vertical blue line in (A and B) shows the intrinsic merit threshold below which no alleles can persist under the AO model (A), but this is not true under the DAA model (B). Indeed, purple arrows show the three alleles with intrinsic merit below this threshold that have positive frequencies at equilibrium in the DAA model only. Alleles that are absent in (B) but supported in (A) are illustrated by the gray color gradient in the negative range of (B); however, for both models there is a strong correlation between the intrinsic merit of a persisting allele and its frequency at equilibrium, as both homozygotes and, on average, heterozygotes carrying high intrinsic merit alleles have a higher fitness than those carrying alleles with lower intrinsic merit (see Equation 9 for the AO model).

The intrinsic merits of persisting alleles are qualitatively different between the AO and DAA model. Under the AO model, all alleles above a threshold intrinsic merit, $w_{thr}$, persisted at equilibrium, for example, as shown in Figure 2A for scenario 62 (where $w_{thr}=0.865$, blue vertical line), while all other alleles were absent at equilibrium. However, under the DAA model, some alleles with intrinsic merits above the threshold may be absent at equilibrium (shown for the same iteration as gray-hatched rectangles in Figure 2B), while alleles with lower intrinsic merits can persist (indicated by purple arrows). Although the intrinsic merit of the “worst” allele is only 0.785, it persists because it is sufficiently divergent that it has a high overdominance, $h_{i}=27.1\text{\%}$, and so its marginal fitness is the same as the “best” allele, whose intrinsic merit is 0.995 and $h_{i}=0.5\%$.

**Figure 3:**
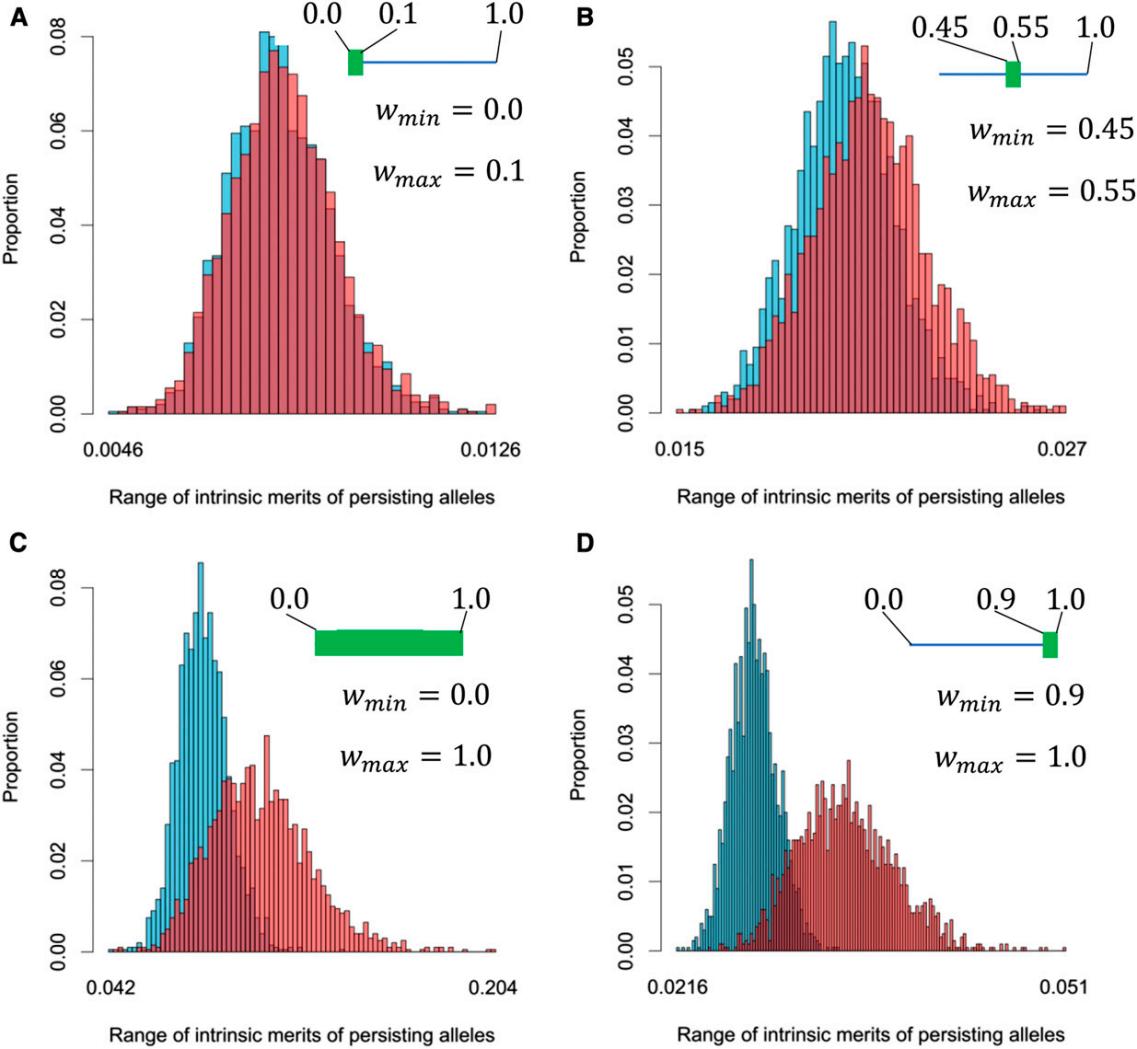
The range of intrinsic merits of the set of alleles maintained at equilibrium for the Random experiment and (A) scenario 3, *i.e.*, $\left(w_{min},w_{max},f,n_{ini}\right)=$(0.0, 0.1, 2, 250); (B) scenario 13, *i.e.*, $\left(w_{min},w_{max},f,n_{ini}\right)$ = (0.45, 0.55, 2, 250); (C) scenario 63, *i.e.*, $\left(w_{min},w_{max},f,n_{ini}\right)=$ (0.0, 1.0, 2, 250); and (D) scenario 73, *i.e.*, $\left(w_{min},w_{max},f,n_{ini}\right)=$ (0.9, 1.0, 2, 250). The red bars correspond to the Divergent Allele Advantage (DAA) model, while the blue bars correspond to the asymmetric overdominance (AO) model. The distribution of the ranges of intrinsic merits for the DAA model changes toward larger ranges relative to the AO model from (A), a scenario where both the initial intrinsic merit range and the maximum intrinsic merit are low, to (D), a scenario where the initial intrinsic merit range is low but the maximum intrinsic merit is high.

In general, the DAA model allows a larger intrinsic merit range for alleles at equilibrium than the AO model. For the effectively infinite population, of the 80 scenarios, more iterations almost always (79 out of 80 for Random and 70 out of 80 for Fixed) had a greater range under the DAA model than the AO model, and on average for each scenario over four times as many individual iterations of the DAA model had a greater range than the AO model (Table 2), with the range on average 6.14% higher for the Random experiment and 5.79% higher for the Fixed experiment (Tables S1 and S2, respectively). The improvement in range afforded by the DAA model is greater for higher maximum fitnesses (see Figure 3 for an example, and Tables S1 and S2 for details), which also correspond to the cases where overdominance of the populations at equilibrium was lower (Table 3).

**Table 2:** Comparison of intrinsic merit ranges and number of alleles between DAA and AO models for both experiments

		DAA (%)	AO (%)	Equal (%)
Random experiment	Wider intrinsic merit range	53.9	12.7	33.3
	More alleles	25.9	37.1	37.1
Fixed experiment	Wider intrinsic merit range	60.0	14.4	25.7
	More alleles	28.2	34.6	37.3

AO, asymmetric overdominance; DAA, Divergent Allele Advantage.

**Table 3:** Average overdominance for different scenarios

Scenarios	$w_{min}$	$w_{max}$	Overdominance ($\bar{h}$) (%)
1–10	0	0.1	∼90
11–20	0.45	0.55	∼45
21–30	0.2	0.6	∼40
31–40	0.3	0.7	∼30
41–50	0.4	0.8	∼20
51–60	0.1	0.9	∼10
61–70	0.0	1.0	< 5
71–80	0.9	1.0	< 1

Approximate average values for the resulting overdominance at equilibrium, given by the increase in the average fitness of heterozygotes compared to the average fitness of homozygotes, for groups of scenarios. Overdominance ($\bar{h}$) was an emergent property of the model; nevertheless, parameters were chosen to cover the range of published estimates for $\bar{h}$ [or the selection coefficient acting on Major Histocompatibility Complex (MHC) genes, s, with $\bar{h}=\frac{s}{1-s}$], which varies with the MHC locus and range from $\bar{h}<5\%$ (Hill *et al.* 1991; Satta *et al.* 1994) to $\bar{h}=85\%$ (Hedrick 1994; Black and Hedrick 1997); all data on humans.

**Figure 4:**
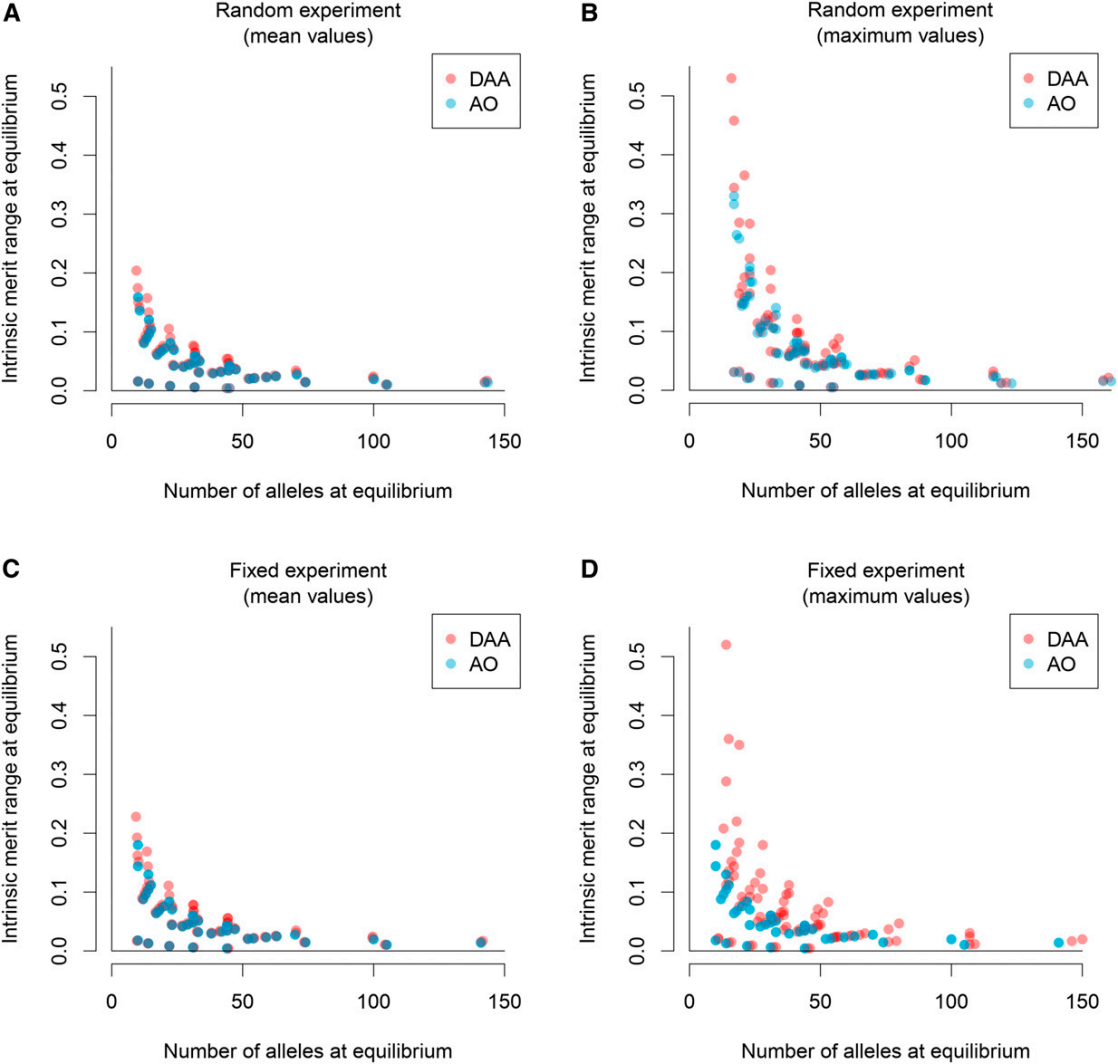
Allele intrinsic merit range at equilibrium for the Random experiment (A and B) and the Fixed experiment (C and D). Mean values (A and C) and maximum values (B and D) of ranges of intrinsic merits of extant alleles at equilibrium, averaged over all scenarios shown *vs.* number of persisting alleles for the Divergent Allele Advantage (DAA) (red) and the asymmetric overdominance (AO) (blue) model. Both models show the well-established (Maruyama and Nei 1981; Takahata and Nei 1990) trade-off between the number of alleles maintained and the intrinsic merit range maintained. While the number of alleles supported is quite similar in both models, the DAA model has a tendency to support larger allele intrinsic merit ranges. For the Fixed experiment in particular, the maximum values found for intrinsic merit ranges of the DAA model far exceeded the respective maximum values of the AO model, while the overall patterns indicated a trend toward combinations of more supported alleles and higher intrinsic merit ranges for the DAA model (red dots shifted upward and to the right).

The initial number of alleles in the population $\left(n_{ini}\right)$ had the largest effect on the number of persisting alleles, with substantially higher numbers persisting for higher initial $n_{ini}$ (Tables S1 and S2). The average number of persisting alleles was similar for the DAA and AO models, with the AO model containing on average 0.99 and 0.68% more alleles at equilibrium for the two experiments (Tables S1 and S2), and fewer iterations having more alleles for the DAA model (25.9 *vs.* 37.1% for the Random experiment and 28.2 *vs.* 34.6% for the Fixed experiment). Despite this, across the whole range of scenarios the DAA model provides, on average, higher ranges of intrinsic merits for similar numbers of persisting alleles (Figure 4, A and C), and this result is even more pronounced when we look at the maximum numbers of alleles and maximum intrinsic merit ranges provided across the iterations for a scenario (Figure 4, B and D).

The weighted mean overlap $\bar{g}=\sum_{i=1}^{k}p_{i}\cdot g_{i}$ (with the allele proportions as weights) of the persisting set of alleles was higher in the AO model than the DAA model for 96.25 (Random experiment) and 87.5% (Fixed experiment) of all scenarios (see Tables S5 and S6), respectively. In particular, alleles with a low average overlap preferentially persist: the mean of the average overlap for the allele with the smallest overlap to the other alleles in the gene pool was higher for the AO model in > 90% of all scenarios (97.5 and 91.25% in the Random and Fixed experiments, respectively). We obtained similar results for alleles on the 1, 2, 5, and 10% percentiles in terms of average overlap (see Tables S5 and S6 for more details), although for higher percentiles the differences between the models became less pronounced. The differences between the AO and DAA models, while seemingly low, were still meaningful, as they only stem from the initial set of alleles without any mutation involved. Differences were most pronounced in the scenarios with low overdominance at equilibrium (scenarios 61–80, see Tables S5 and S6), where the efficiency of the DAA model, also in terms of intrinsic merit range afforded, was greatest.

## Discussion

The mechanisms underpinning the extreme polymorphism at the MHC have remained a much-debated and open question for decades. The most recent explanations for this phenomenon center on the DAA hypothesis, which proposes that heterozygotes with more divergent alleles allow for broader antigen presentation to immune cells (Wakeland *et al.* 1990). Validating this hypothesis would answer a long-standing question in evolutionary biology, while also being of significant practical value, providing mechanisms that could be exploited to improve the health of livestock and managed wildlife populations. Our simple model, allowing for differential recognition of peptides by different alleles, naturally supports more divergent alleles in the population, providing a first quantitative demonstration of DAA as a key driver of MHC polymorphism.

We couched alternative models within a single, general framework and used this to compare AO with a new model based on the DAA hypothesis, based on the idea that differences in the antigen-binding site between alleles are influenced by selection (Erickson *et al.* 2001). Key to our results was that the DAA model behaved differently to traditional AO models, because alleles with low intrinsic merit may survive if they complement the most common alleles in the gene pool. Therefore, the model supported significantly larger ranges of intrinsic allele merits, while maintaining similar numbers of alleles because of the higher overdominance of the less intrinsically fit alleles. This is an important advance on earlier studies, which have typically found that the maintenance of large numbers of alleles required a narrow window for the intrinsic merits of the persisting alleles (De Boer *et al.* 2004). Therefore, our results alleviate concerns about the capacity for overdominance in general to maintain both larger numbers of alleles and variation in the intrinsic merit of these alleles.

The assumption underpinning the DAA hypothesis is that the fitness of a genotype increases as the alleles at a locus cover more of the immune response void, *i.e.*, the combined immune response defects present (Wakeland *et al.* 1990). In the DAA model presented here, this can be achieved if the overlap $g_{ij}$ (see above) between any two alleles $A_{i}$ and $A_{j}$ is as small as possible, so that these alleles recognize largely distinct pathogen epitopes. Therefore, one would expect a tendency to minimize the overlap between alleles from any DAA model. This is indeed the case in our model: despite the relatively low number of repetitions, the overlap was (often significantly) lower in the DAA model compared to the AO model in the vast majority of scenarios. These results also show substantial divergence between extant alleles, replicating a feature detected in a large number of studies (Richman *et al.* 2001; Babik *et al.* 2008; Eizaguirre *et al.* 2012; Ellison *et al.* 2012; Lenz *et al.* 2013; Pierini and Lenz 2018), which provide empirical support for the DAA model.

The larger intrinsic merit ranges in the DAA model, together with the observations that overlap between the alleles in the gene pool decreases and allele numbers at equilibrium increase with increasing $n_{ini}$ (the initial number of alleles), imply that, over time, a population may evolve to a state where the gene pool consists of alleles that have a high degree of complementarity, via a process where lower overlap between alleles can subsequently result in wider intrinsic merit ranges, and even larger equilibrium allele numbers.

Our results complement recent work (Pierini and Lenz 2018) that demonstrates that MHC heterozygotes with more genetically divergent alleles do bind more peptides and have higher frequency in the population, suggesting that they have higher fitness. Our results show that a simple peptide-recognition model that mirrors these results is sufficient on its own to maintain low intrinsic merit alleles in the population. Together, these results indicate that DAA could be an important driver of MHC polymorphism, predict the presence of relatively poor alleles (alleles with low intrinsic merits) in the gene pool, and thereby explain the wide number of associations of MHC alleles with disease. The practical application of these results is to provide a better way to identify the quality of heterozygotes, in particular those with enhanced pathogen recognition, through measurement of their genetic divergence. Breeding strategies based on these techniques (selecting animals with a set of highly divergent alleles) should in turn allow us to improve the disease resistance of managed animals, including wild animals of conservation concern. The ability to identify MHC genotypes that increase susceptibility to infectious and parasitic diseases simplifies personalized medicine, and allows us to focus resources on individuals at increased risk of infection.
